# Effects of Laser Powers on Microstructures and Mechanical Properties of Al_0.5_FeCoCrNi High-Entropy Alloys Fabricated by Laser Melting Deposition

**DOI:** 10.3390/ma15082894

**Published:** 2022-04-15

**Authors:** Xuesong Zhang, Yinbao Tian, Sunusi Marwana Manladan, Yan Cui, Keping Geng, Yangchuan Cai, Jian Han

**Affiliations:** 1 School of Materials Science and Engineering, Tianjin University of Technology, Tianjin 300384, China; zhangxs9283@126.com (X.Z.); tianyinbao@email.tjut.edu.cn (Y.T.); 2 School of Materials Science and Engineering, Tianjin University, Tianjin 300072, China; smmanladan@tju.edu.cn (S.M.M.); dianleyan@163.com (Y.C.); 3 Department of Mechanical Engineering, Faculty of Engineering, Bayero University, Kano 3011, Nigeria; 4 School of Mechanical Engineering, Tianjin Sino-German University of Applied Sciences, Tianjin 300350, China; gengkeping@tsguas.edu.cn

**Keywords:** high-entropy alloys, laser melting deposition, laser power, microstructure, mechanical properties, strengthening mechanism

## Abstract

High-entropy alloys (HEAs) show great promise for various applications in many fields. However, it still remains a challenge to obtain the ideal match of the tensile strength and the ductility. In this paper, Al_0.5_FeCoCrNi walls were fabricated through laser melting deposition (LMD) technology with laser power ranging from 1000 W to 1800 W. Along with the increase in laser power, the average size of the Al_0.5_FeCoCrNi walls increased from 14.31 μm to 34.88 μm, and the B2 phase decreased from 16.5% to 2.1%. Notably, the ultimate tensile strength and the ductility of the 1000 W bottom wall were 737 MPa and 24.6%, respectively, while those of 1800 W top wall were 641 MPa and 27.6%, respectively, demonstrating that the tensile strength of the walls decreased and the ductility increased with the increase in laser power. Furthermore, quantitative calculation revealed that grain boundary strengthening and dislocation strengthening were the two major forms of strengthening compared to the others. This study concluded that the mechanical properties of HEAs could be regulated by laser power, enabling broader applications in industry with favorable tensile strength or ductility.

## 1. Introduction

High-entropy alloys are composed of at least five principal metallic elements with the fractions of each principal element ranging from 5–35 at.% [1,2]. Due to their advantages in many aspects, such as high strength, fatigue and fracture resistance [3,4,5], thermal stability [6], wear resistance [7], corrosion resistance [8], and some novel physical properties, HEAs have application prospects in a variety of engineering applications. Al_x_CoCrFeNi is a widely studied alloy system with a dual phase including face-centered cubic (FCC) and body-centered cubic (BCC) structures [9,10,11]. Furthermore, its lattice distortion worsens with increased Al content, and the FCC phase gradually becomes unstable, changing to BCC/B2. Consequently, the strength increases while the ductility decreases. FeCoCrNi HEA consists mainly of the FCC phase, and it has excellent ductility (50%) and insufficient strength (457 MPa) [12]. AlCoCrFeNi HEA is dominated by the BCC/B2 phase, with a strength of about 1074 MPa and limited ductility of only 1.2% [13,14]. As a consequence of integrating the benefits of FCC and BCC/B2 phases, balanced mechanical qualities of strength-toughness matching may be obtained.

In recent years, many researchers have studied Al_0.5_FeCoCrNi HEAs because of their dual-phase structure, facilitating an increase in potential applications [10,11,15,16]. Niu et al. [15] prepared Al_0.5_CoCrFeNi ingots with nominal composition by arc melting. They concluded that the excellent tensile properties of the heat-treated Al_0.5_CoCrFeNi HEA can be attributed to the nanosized B2 phase in the interdendritic region and the precipitates in the dendritic region. More BCC phases were responsible for increased strength after 1 h heat treatment. Lin et al. [17] investigated the microstructure and hardness properties of as-cast Al_0.5_CoCrFeNi alloy. They found the as-cast high-entropy Al_0.5_CoCrFeNi alloy to have an FCC solid solution structure, whereas the alloys aged at 350–950 °C had an (CC + BCC solid solution structure. The hardness of the Al_0.5_CoCrFeNi alloy was increased after aging. Therefore, according to the above research, phase transition provides the possibility to optimize mechanical performance.

However, the Al_0.5_CoCrFeNi HEAs involved in current research are mainly produced by casting. In the casting process, it is difficult to obtain a relatively uniform and fine microstructure in the ingot [18,19,20]. Compared to traditional manufacturing processes, additive manufacturing, as an emerging technology, can provide great flexibility for the design and manufacture of components. Furthermore, laser melting deposition (LMD) technology is characterized by its ultrafast heating and cooling rates [21], which can prevent the formation of intermetallic compounds and the diffusion of constituent elements. Thus, a refined microstructure can be produced to improve the mechanical properties of manufactured products. Due to the enhanced quality and mechanical performance, LMD technology has increasingly been used to manufacture HEAs [22,23,24]. A number of researchers presented studies on the additive manufacturing of HEAs. Brif et al. [12] produced an FeCoCrNi HEA with satisfactory strength and ductility by selective laser melting (SLM). Kunce et al. [25] selected different laser scanning rates in the laser-engineered net shaping (LENS) process to explore the effects of cooling rates on the HEA microstructure. It was found that, as the laser scanning rate increased, the average grain size decreased. The average microhardness of the AlCoCrFeNi alloy produced by LENS was about 13% higher than that of the as-cast alloy. The high hardness could be attributed to the grain refinement from the LENS process. Zhou et al. [16] fabricated the Al_0.5_FeCoCrNi HEA using SLM with gas-atomized pre-alloy powders. After the SLM treatment, the BCC phase in the powder transformed into the FCC phase. The samples exhibited excellent tensile properties, i.e., yield strength and ultimate tensile strength of 579 MPa and 721 MPa, respectively.

These studies illustrate that additive manufacturing is a promising solution for the fabrication of HEAs. The phase fractions of Al_0.5_FeCoCrNi HEA can be controlled through the adjustment of processing parameters, such as laser power in additive manufacturing. So far, no research has been reported on the manufacture of Al_0.5_FeCoCrNi HEAs by LMD. In this study, the influence of laser power (1000 W, 1400 W, 1800 W), on the microstructure and mechanical properties of the Al_0.5_FeCoCrNi HEA manufactured by LMD was investigated. Furthermore, the strengthening contributions from different mechanisms was precisely assessed. This study provides new insight for preparing the optimized dual-phase HEA using LMD.

## 2. Materials and Methods

### 2.1. Materials

**Figure 1:**
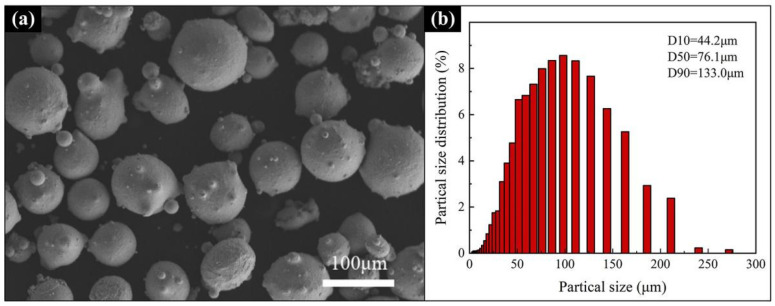
Al_0.5_FeCoCrNi HEA powders: (**a**) SEM microstructure; (**b**) particle size distribution (DX is the particle size when the cumulative distribution is X%).

The material used as the substrate in this study was 316 stainless steels (120 × 40 × 10 mm^3^). The chemical composition of the steel is listed in Table 1. The LMD process used powder as raw material. Therefore, the pre-alloyed powders in this experiment were prepared by nitrogen atomization. The designed molar ratio of the Al_0.5_FeCoCrNi HEA powder was 0.5:1:1:1:1, and the powder purity was 99.0–99.5%. The chemical composition of the Al_0.5_FeCoCrNi HEA powders is listed in Table 2. Figure 1a shows the SEM image of the Al_0.5_FeCoCrNi-HEA raw powders. Obviously, the powder particles were spherical, which was beneficial to fluidity during the powder feeding process. As shown in Figure 1b, the particle size distribution of the Al_0.5_FeCoCrNi-HEA powder ranged from 45 to 150 μm, with an average particle size of ~122 μm.

### 2.2. Fabrication of Al_0.5_FeCoCrNi-HEA Wall by LMD

**Figure 2:**
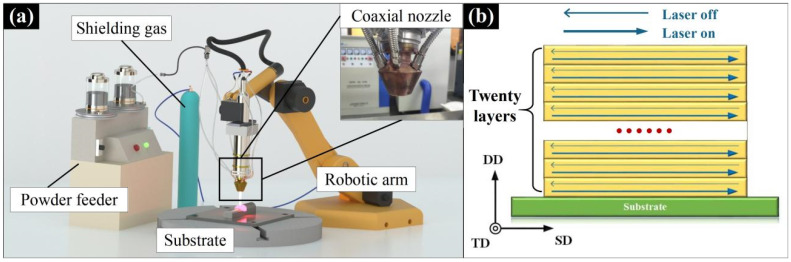
(**a**) Schematic diagram of the LMD equipment; (**b**) schematic diagram of the scanning strategy. SD (laser scanning direction), DD (deposition direction), TD (transverse direction).

The surface of the 316 stainless steel was ground using a milling machine, and then cleaned with acetone solution. An HANSGS-RJ0016-F3K system (Han’s Laser Technology Industry Group Co., Ltd., Shenzhen, China) was used to fabricate the Al_0.5_FeCoCrNi HEA wall. Figure 2a shows the schematic diagram of the LMD equipment. The LMD system mainly consisted of a PLC S7-1200 master control system (Siemens Industry Group Co., Ltd., Shanghai, China), a PLC S7-1200 fiber laser (Han’s Laser Technology Industry Group Co., Ltd., Shenzhen, China), a KR22 R1610 robot arm (KUKA Robotics Manufacturing China Co., Ltd., Shanghai, China), a SF-02 coaxial powder feeding system (Han’s Laser Technology Industry Group Co., Ltd., Shenzhen, China), and an argon chamber. In this experiment, three different laser powers (1000 W, 1400 W, and 1800 W) were used to produce the Al_0.5_FeCoCrNi HEA walls. Figure 2b is a schematic diagram of the LMD process, in which a single scanning strategy was used. After each layer was scanned by the laser, the height of the laser head was raised by a distance of 1 mm, and the number of scanned layers was 20. During the whole scanning process, the laser moved at a scanning speed of 300 mm·min^−1^. The powder was fed into the molten pool through a coaxial nozzle by a closed-loop powder feeding device at a speed of 2 rpm. Throughout the scanning process, the shielding gas (argon) flow rate used was kept at 5 nL·min^−1^ to ensure that the oxygen content was limited to less than 10 ppm. Two walls were fabricated for each laser power. It should be noted that the process parameters were based on our previous research results [26].

### 2.3. Microstructure Characterization and Mechanical Properties

**Figure 3:**
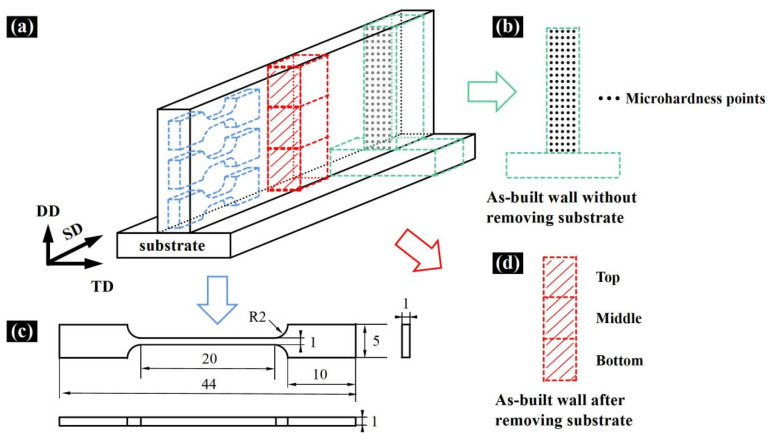
Schematic diagram of sample location and size: (**a**) sampling location; (**b**) microhardness sample; (**c**) tensile sample (in mm); (**d**) OM and XRD samples.

As shown in Figure 3, the samples for the characterization of microstructural and mechanical properties were taken from specified locations in the as-deposited Al_0.5_FeCoCrNi HEA. To observe the phase composition and grain growth trend in the HEA, the samples were cut from the bottom, middle, and top locations of the HEA manufactured by LMD, as illustrated in Figure 3d. The samples were subjected to grinding and polishing according to the standard procedures of metallography. The polished samples were etched in aqua regia (the ratio of nitric acid to hydrochloric acid was 1:3) for 15 s. The DSX-M3 optical microscope (OM, Olympus Co., Ltd., Tokyo, Japan) was used to observe the microstructure. The phase identification was carried out using a SmartLab advanced X-ray diffractometer (Rigaku D/max/2500PC, Hitachi Co., Ltd., Tokyo, Japan) at a scanning speed of 4°·min^−1^ and a range of 20° to 100°. The analysis was performed using Jade 6.5 software. In order to further characterize the microstructure, an FEI TECNAI F30 transmission electron microscope (TEM, FEI Co., Ltd., Hillsboro, OR, USA) was used to obtain high-magnification electron images and microscopic selected area electron diffraction (SAED) patterns.

The microhardness of the Al_0.5_FeCoCrNi sample was measured by the HMV-2T microhardness tester; the sampling location is shown in Figure 3b. From the substrate to the top of the sample, the distance between two adjacent indentation points was 1 mm. At the same height, five different measurements were taken to obtain the average hardness. In order to prevent the influence of work hardening on the hardness, the minimum distance between the indentation points was 50 μm. The applied load was 2.942 N, and the loading time was 15 s.

An MTS SANS CMT5105 testing machine was used to conduct uniaxial tensile tests on the samples obtained from the bottom, middle, and top locations of the HEA at room temperature. The dimensions of the tensile test specimen are shown in Figure 3c, and the strain rate was 10^−3^ s^−1^. An S-3400N scanning electron microscope (SEM, Hitachi Co., Ltd., Tokyo, Japan) was used to observe the fracture of post-test tensile samples.

## 3. Results and Discussion

### 3.1. The Phases and Microstructure of the Al_0.5_FeCoCrNi HEA Manufactured by LMD

**Figure 4:**
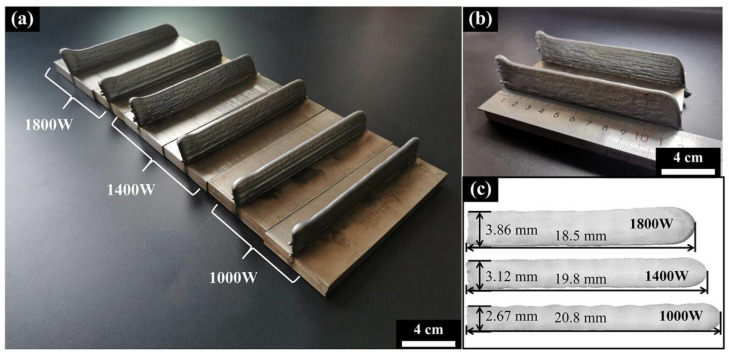
The Al_0.5_FeCoCrNi wall manufactured by LMD: (**a**) macrostructure; (**b**,**c**) specimen dimensions.

Figure 4 shows the macrostructure of the Al_0.5_FeCoCrNi samples produced using different laser powers. Generally, all the samples had good quality without obvious defects such as pits and splashes. The dimensions of the specimen manufactured by LMD are shown in Figure 4b,c. Here, the dimensions of the specimen were changed with increasing laser power. As the laser power increased, the height of the specimen increased while the width decreased. Wen et al. studied and explained this phenomenon [27]. The width and thickness of the sample are closely related to the absorbed laser energy in the additive manufacturing process. The width of the specimen increases with increased laser energy. At the same time, the height of the wall is determined by the overall cross-section and width of each layer. If the layer is wider, the sample becomes shorter. 

**Figure 5:**
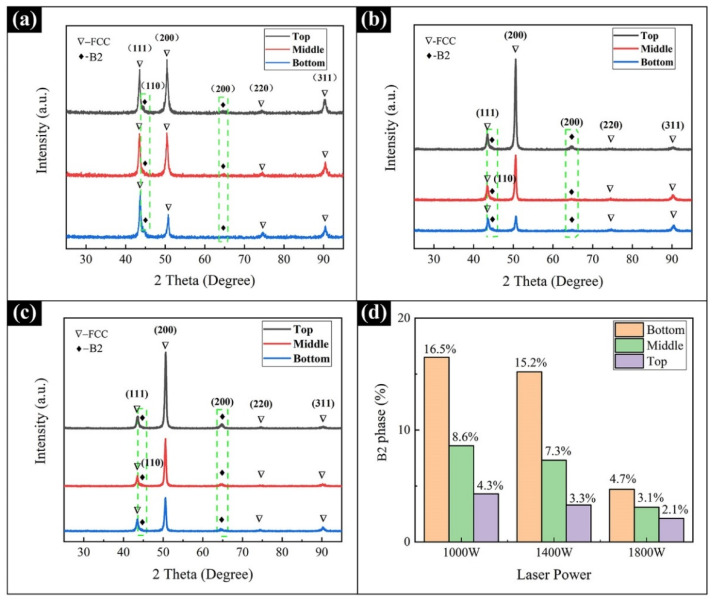
The XRD results of the Al_0.5_FeCoCrNi HEA manufactured by LMD with different laser powers: (**a**) 1000 W; (**b**) 1400 W; (**c**) 1800 W. (**d**) Fractions of B2 phase in different locations.

The phase structure of three Al_0.5_FeCoCrNi samples were qualitatively characterized by XRD. Figure 5a-c show the XRD results of the bottom, middle, and top locations of the samples produced with the laser powers of 1000 W, 1400 W, and 1800 W, respectively. It can be seen from Figure 5 that there are two types of solid solution phases in the Al_0.5_FeCoCrNi sample, i.e., FCC and B2, which is consistent with the results of previous studies [15]. On the one hand, for the sample produced with 1000 W laser power, as shown in Figure 5a, at the bottom of sample, the intensity of the (111) peak was much higher than that of the (200) peak. However, as the height of the sample increased, the intensity of the (200) peak gradually increased and eventually exceeded that of the (111) peak, indicating a preferred orientation in the sample. This phenomenon can also be observed in the samples produced with 1400 W and 1800 W. On the other hand, it can also be seen from Figure 5 that, for the same location (bottom, middle, or top) in three samples, the intensity of the (200) peak gradually improved with the increase in laser power, also indicating a preferred orientation in the sample. The phenomenon of preferred orientation is very common in the LMD process, because the growth of crystal grains tends along the maximum temperature gradient in the deposition direction [28,29]. Figure 5d shows the relationship linking the percentage of B2 phase, laser power, and sample locations. For a specified laser power (1000 W, 1400 W, or 1800 W), as the height of the sample increased, the B2 phase in the sample gradually decreased. The highest B2 phase was obtained in the bottom location of the sample, followed by the middle location and then the top location. Furthermore, for the same location (bottom, middle or top) in the three samples, the B2 phase amount also changed with the laser power. In sum, the B2 content produced at 1000 W power was significantly higher than that produced at 1800 W. This is because an increase in power resulted in a higher temperature in the sample. At higher temperatures, the B2 phase is not as stable as the FCC phase, leading to a phase transition from B2 to FCC [10,30].

**Figure 6:**
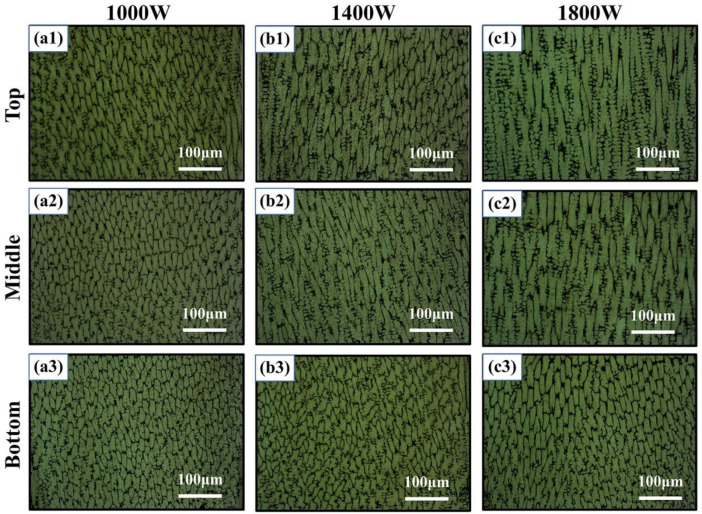
The microstructures of the Al_0.5_FeCoCrNi HEA fabricated by LMD (**a1**–**c3**), where 1, 2, and 3 represent the top, middle, and bottom locations of the sample, respectively.

**Figure 7:**
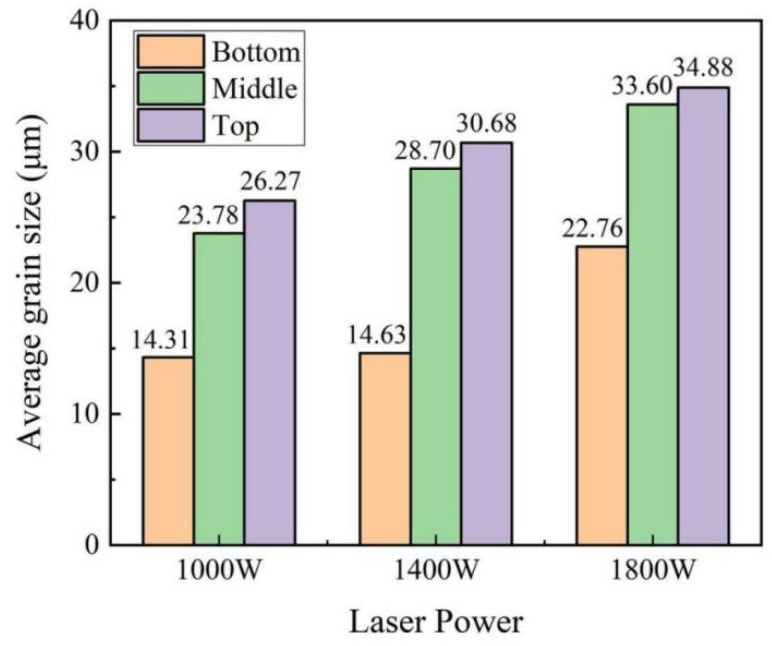
Average grain size of the Al_0.5_FeCoCrNi HEA manufactured by LMD.

The microstructure of different locations of the HEA samples manufactured by LMD with different laser powers is shown in Figure 6. Figure 7 shows the average grain size in the different locations of the samples with varied laser powers. Generally, the microstructures of the samples mainly exhibited columnar crystals. On the one hand, as the height of the sample increased during the deposition process, heat gradually accumulated, thereby driving the growth of grains, and crystal grains became longer. The average grain size of the bottom location was significantly smaller than that of the middle and top locations. However, there was almost no difference between the average grain size of the middle and top locations, as shown in Figure 7. Moreover, it can also be seen that, for the same location (bottom, middle, or top) in the three samples, the grain size increased with laser power. At higher laser power, the size of the crystal grains was relatively larger, and columnar grains tended to be much longer. It should be pointed out that the columnar crystals here were mainly composed of the FCC phase, while the B2 phase was mainly distributed at the grain boundaries with a very small content, as proven in Section 3.3.

The solidification behavior in the LMD process can be explained by the nonequilibrium solidification theory and constitutional undercooling theory [28]. In this process, the growth behavior of crystal grains is mainly determined by the liquid temperature gradient G and the solidification rate R. G and R control the undercooling conditions and determine the front solidification instability and grain morphology during solidification [31,32]. During the solidification process, G is highest at the bottom of the molten pool, and R is highest at the surface of the molten pool. In the whole LMD process, the G/R value of the Al_0.5_FeCoCrNi sample was in the range favorable for the growth of columnar crystals [24,33]. As known to us, a suitable constitutive supercooled zone is formed in the solidification frontier, which induces the growth of columnar crystals. The wall is formed by overlapping adjacent layers. A new layer would cause a certain volume of the previous layer to be melted. Therefore, the crystal grains at the top of the melting pool are melted, and the columnar grains of the previous layer continue to grow epitaxially. With the increased height of the sample manufactured by LMD, the heat generated by the laser continuously accumulates, thereby ensuring continuous growth of the columnar crystals [29]. Therefore, it is reasonable for the columnar crystals to nucleate and grow in the wall.

### 3.2. Mechanical Properties

**Figure 8:**
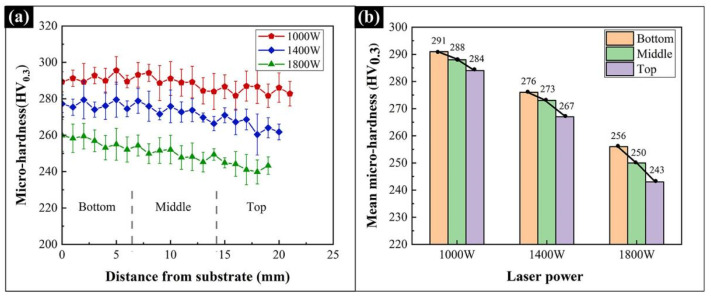
The hardness of the Al_0.5_FeCoCrNi HEA manufactured by LMD at different powers and locations: (**a**) hardness distribution; (**b**) average hardness.

Figure 8a,b respectively show the hardness distribution and average hardness of different locations in the Al_0.5_FeCoCrNi samples at different laser powers. As shown in Figure 8a, as the height of the sample improved, the hardness decreased gradually. At all laser powers, the bottom locations exhibited the highest hardness, followed by the middle locations and then the top locations. As shown in Figure 8b, from the bottom to the top locations, the Vickers hardness of the 1000 W sample dropped from 291 HV_0.3_ to 284 HV_0.3_, the Vickers hardness of the 1400 W sample dropped from 276 HV_0.3_ to 265 HV_0.3_, and the Vickers hardness of the 1800 W sample dropped from 256 HV_0.3_ to 243 HV_0.3_. The smallest drop in hardness was obtained in the sample at 1000 W, while the largest one was obtained in the sample at 1800 W. Furthermore, for the same location, the microhardness of the sample decreased with increased laser power.

**Figure 9:**
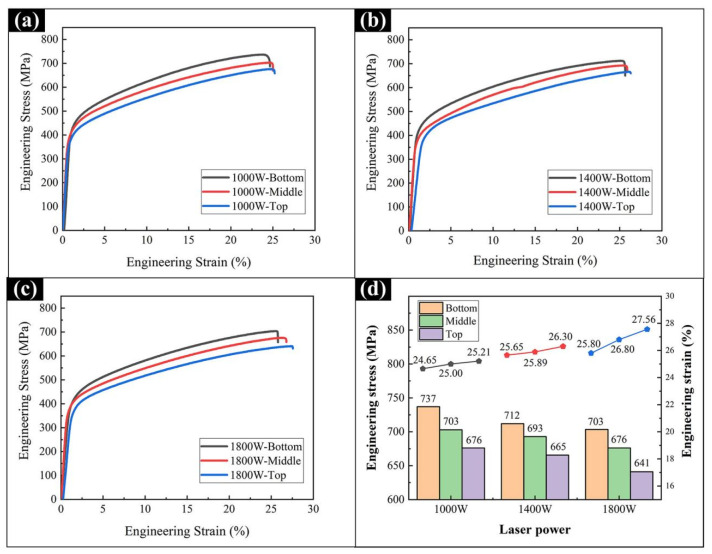
The tensile stress-strain curves of the Al_0.5_FeCoCrNi HEA samples manufactured by LMD at different powers and different locations: (**a**) 1000 W; (**b**) 1400 W; (**c**) 1800 W. (**d**) Average strength and strain.

**Figure 10:**
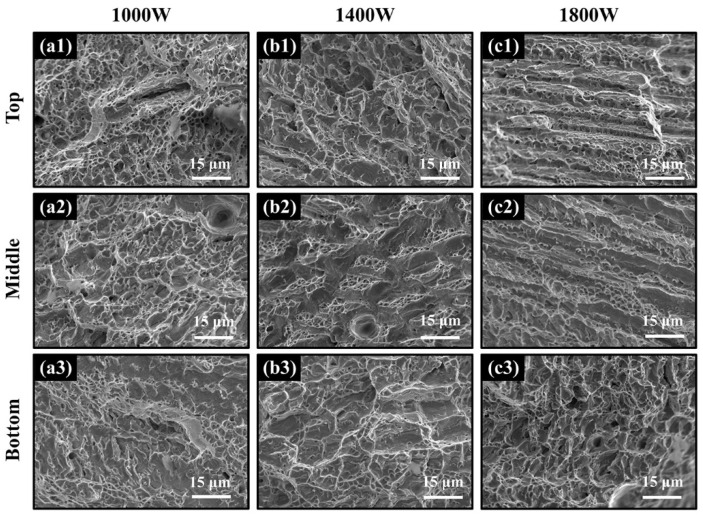
The fracture surfaces of the Al_0.5_FeCoCrNi HEA fabricated by LMD (**a1**–**c3**), where 1, 2, and 3 represent the top, middle, and bottom locations of the sample, respectively.

Figure 9 shows the tensile properties of the Al_0.5_FeCoCrNi samples at different powers and locations. In general, as the laser power increased, the strength of the sample gradually decreased, and the ductility improved slightly. Moreover, at the same laser power, the tensile properties changed significantly with increasing height of the sample. Compared with the top location, the bottom location showed better strength and a slight decrease in ductility. Figure 10 shows the tensile fracture morphology of the samples at different powers and different locations. Generally, the fracture surfaces exhibited typical ductile fracture characteristics. The well-observed dimples, which were numerous and dense, indicated good ductility. Due to the large number of columnar crystals produced in the additive manufacturing process, Figure 10 shows obvious columnar crystal fracture morphology, which is common in columnar crystals manufactured by LMD [34]. In the morphology of the entire dimples, no obvious spherical impurities could be observed, proving that there was no uneven melting of powder in the deposition process.

### 3.3. Strengthening Mechanisms in the Al_0.5_FeCoCrNi HEA Manufactured by LMD

Combined with the microstructures, it could be deduced that the yield strength of HEA is attributed to a combination of friction stress, solid solution strengthening, dislocation strengthening, and grain boundary strengthening. In order to qualify the strengthening mechanisms, the following microstructure-related equation was utilized:

(1) $$\sigma_{y}=\sigma_{0}+\sigma_{SS}+\sigma_{GB}+\sigma_{DS}$$

where $\sigma_{0}$, $\sigma_{SS}$, $\sigma_{GB}$, and $\sigma_{DS}$ represent yield stresses resulting from the friction stress, solid solution strengthening, grain boundary strengthening, and dislocation strengthening, respectively.

The friction stress is the intrinsic lattice resistance to dislocation motion. The $\sigma_{0}$ value here is the resistance from a complex lattice of all these five constituent atoms rather than that from the parent material. For the recently emerged HEAs, the relevant study is still not systematic and clear enough. Moreover, the values needed here for the Al_0.5_FeCoCrNi HEA are not available in the literature. Hence, similar to the approximations made in [35], the rule of mixtures was used to estimate the friction stress, which was calculated as 95 MPa.

As for the solute atoms, they are normally discussed in dilute solid solution alloys with a single base metal, plus a certain number of interstitial and substitutional solute atoms. These interstitial or substitutional solute atoms generate a local stress field which hinders the dislocation motion and, therefore, strengthens the alloy. Hence, the current HEA can be treated as a CrFeCoNi solvent matrix containing Al solutes. A standard model for substitutional solid solution strengthening based on elastic dislocation solute interactions can be used to estimate the potency of solid solution strengthening [36].

(2) $$\sigma_{ss}=M\times \frac{G_{M}\varepsilon_{s}^{1.5}c^{0.5}}{700},$$

where $G_{M}$ = 84 GPa is the shear modulus of the CrFeCoNi matrix [37], c is the atomic ratio of Al in the FCC matrix, and M = 3.06 is the Taylor factor [38]. The interaction parameter $\varepsilon_{s}$ = 16.4 was referenced in [39]. Thus, the strength enhancement caused by the solid solution strengthening in the current HEA was about 44.6 MPa.

It is obvious that there was a dual-phase structure in the Al_0.5_FeCoCrNi HEA manufactured by LMD. Furthermore, the laser power had an impact on the phase composition and grain size of the current alloy. Therefore, for grain boundary strengthening and dislocation strengthening, it is needed to discuss the contributions of the FCC phase and the B2 phase, respectively.

The mechanical test results show that the Al_0.5_FeCoCrNi samples produced by LMD had good mechanical properties. In addition to the type of material, the preparation method also affects the mechanical properties [40]. The microstructure in Figure 6 shows that the grain size was very small, which is because LMD has a fast solidification rate (10^3^–10^8^ K·s^−1^) and a large degree of undercooling [23]. A smaller grain size yields more grain boundaries per unit volume, which helps to release more dislocations during plastic deformation. In this case, it is difficult to have local stress concentration, and the possibility of cracking is, therefore, reduced significantly. As a result, the metal can resist a large amount of deformation and exhibit high plasticity. Unique fine-grain strengthening is one of the major reasons for the high strength of materials produced by additive manufacturing [24,40]. The high strength of the Al_0.5_FeCoCrNi HEA follows the Hall-Petch equation, which quantitatively describes the increase in yield strength of polycrystalline materials as the grain size decreases [41,42]. The yield strength can be expressed using a Hall-Petch relationship as given below.

(3) $$\sigma_{GB}=K_{FCC}d^{0.5}V_{FCC}+K_{B2}d^{0.5}V_{B2},$$

where d is the average grain size, $V_{FCC}$ and $V_{B2}$ are the volume fractions of the two phases, K is the Hall-Petch coefficient depending on the grain boundary structure. Based on Equation (3), the Hall-Petch coefficients, $K_{FCC}$ and $K_{B2}$ were taken as 464 and 1565 MPa∙μm^0.5^ for FCC and B2 phases, respectively [43,44]. The columnar crystals were mainly composed of FCC phase, while the B2 phase was mainly distributed at grain boundaries. Therefore, the FCC phase and the B2 phase were calculated with the same grain size and distinguished by volume fractions. According to this principle, the stress of the LMD sample corresponded to the grains [24,33]. The calculation results of grain boundary strengthening are listed in Table 3. Therefore, fine-grain strengthening played an important role in the Al_0.5_FeCoCrNi HEA manufactured by LMD. 

Due to the rapid solidification of the LMD process, a large number of dislocations are formed in the crystal structure [40,45,46], which can lead to complex dislocation interactions. When dislocations move, they tend to cross with each other, thereby causing dislocation entanglement. This hinders the movement of dislocations, thereby resisting deformation and, consequently, influencing the strength [40].

**Figure 11:**
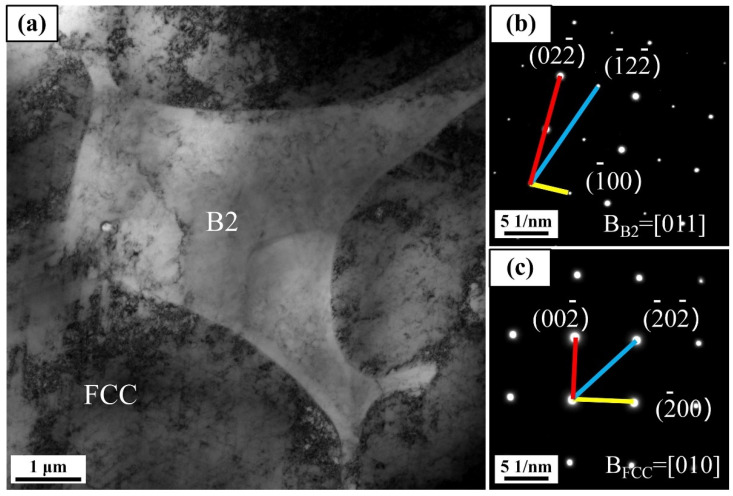
TEM image of Al_0.5_FeCoCrNi HEA manufactured by LMD: (**a**) bright-field TEM image with corresponding SAED pattern; (**b**) SAED pattern of B2 phase; (**c**) SAED pattern of FCC phase.

The reason for the difference in mechanical properties can be explained by the dual-phase structure of the Al_0.5_FeCoCrNi HEA. According to Figure 5, taking a 1000 W sample as an example, the structure of the Al_0.5_FeCoCrNi HEA with FCC and B2 dual phases was further studied. Figure 11 shows the TEM image of the Al_0.5_FeCoCrNi HEA manufactured by LMD. Figure 11b,c show the selected location diffraction (SAED) pattern corresponding to the B2 and FCC phases, respectively. The distribution of FCC and B2 phases is clear. Specifically, after the analysis of interplanar spacing, the accurate phase type of the B2 area in Figure 11a was identified as an ordered B2 phase [47].

In general, FCC-type HEAs are soft and tough, while B2-type HEAs are hard and brittle [48,49]. Both FCC and B2 structures include 12 slip systems. Each sliding surface of the FCC structure has three sliding directions, while each sliding surface of the B2 structure has only two sliding directions. Therefore, for the same material, it is easier for the FCC structure to reach the critical resolved shear stress in plastic deformation than the B2 structure. Furthermore, the atomic packing density of the FCC slip surface is larger and its slip resistance is smaller. Thus, the FCC phase has better ductility. From Figure 5d, we can see that, as the laser power increased, the content of the B2 phase gradually decreased, while the content of the FCC phase gradually increased. Therefore, the presence of a large amount of FCC phase improved the ductility of Al_0.5_FeCoCrNi HEA.

**Figure 12:**
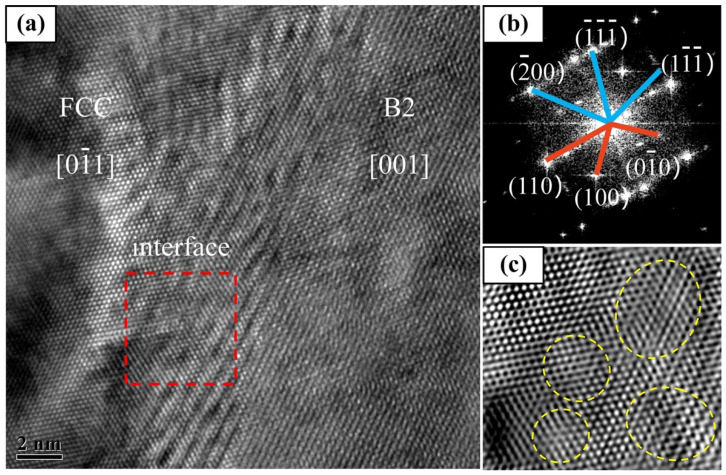
TEM image of Al_0.5_FeCoCrNi HEA manufactured by LMD: (**a**) high-resolution image of the dual-phase interface; (**b**) SAED pattern of dual phase in the high-resolution image; (**c**) local high-resolution image at the dual-phase interface.

Furthermore, the two-phase structure caused the lattice distortion to form an elastic stress field when combined, thereby hindering the movement of dislocations, which would have also increased the strength of the Al_0.5_FeCoCrNi HEA. A high-resolution image of the two-phase interface is shown in Figure 12. Figure 12b shows the diffraction of the B2 [001] crystal axis and FCC [011] crystal axis corresponding to the high-resolution region. Figure 12c shows a higher-resolution image of the region indicated by the red box in the two-phase interface. The atomic arrangement at the junction of the FCC and B2 phases is apparent. In the dual-phase (FCC + B2) HEA, due to the structural differences between the two phases, the deformation and relative movement between the two phases were restricted, and the twisted connection shown in Figure 12c occurred. More energy was often needed for the dislocations and other defects to pass through the joints of the twisted phase. Therefore, the two phases (FCC + B2) further improved the mechanical properties of the Al_0.5_FeCoCrNi HEA due to the difference in the two-phase structure [50].

In crystalline materials, deformation is caused by the movement of dislocations. However, as the dislocations continue to increase, the interaction between them can also hinder the movement of the dislocations. In other words, a large number of dislocations increase the strength of the matrix. According to Taylor hardening model, the relationship can be expressed as follows [51]:

(4) $$\sigma_{DS}=M\alpha Gb\rho^{0.5}.$$

In this study, the laser power has an effect on the composition of the two phases; thus, the Equation (4) can be further expressed as:

(5) $$\sigma_{DS}=\sigma_{DS,FCC}V_{FCC}+\sigma_{DS,B2}V_{B2},$$

where G = 74.06 MPa is the shear modulus [39], $V_{FCC}$ and $V_{B2}$ are the volume fractions of the two phases, b is the magnitude of the Burgers vector, α = 0.2 is a constant [51], and M denotes the Taylor factor. The Taylor factor takes a value of 3.06 in FCC-structured metals and a value of 2.754 in B2-structured metals, respectively [38]. The Burgers vector is $b_{FCC}=\sqrt{2}/2a_{FCC}$ for FCC phase and $b_{B2}=\sqrt{3}/2a_{B2}$ for B2 phase, with a being the lattice constant [52]. The dislocation density ρ can be calculated by the Williamson-Hall equation as follows [53]:

(6) $$\delta_{hkl}\frac{\cos\theta_{hkl}}{\lambda }=\frac{1}{D}+2e\frac{\sin\theta_{hkl}}{\lambda },$$

(7) $$\rho =14.4\frac{e^{2}}{b^{2}},$$

where $\delta_{hkl}$ is the physical broadening of the full width at half maximum (FWHM) of the diffraction peak, e is the microstrain, b is the magnitude of the Burgers vector, D is the apparent crystallite size parameter, θ is the Bragg angle of the analyzed peaks, and λ is the X-ray wavelength, which is equal to 0.154056 nm for Cu-Kα radiation. Background reduction, Kα2 stripping, and data analysis were performed using the MDI Jade 6.5 software. On the basis of Equations (6) and (7), the dislocation density ρ in the Al_0.5_FeCoCrNi HEA manufactured by LMD is estimated in Table 4. Putting the estimated ρ value into Equation (4), the yield stress resulting from the initial dislocations is listed in Table 3.

**Figure 13:**
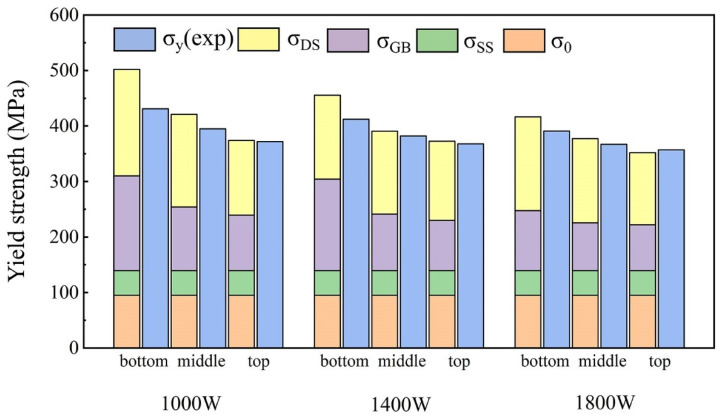
Comparison of theoretical calculations and experimental measurements of yield strength of Al_0.5_FeCoCrNi HEA manufactured by LMD.

In the present HEA, the calculated values of the quantitative contribution from solid solution strengthening, dislocation strengthening, and grain boundary strengthening are listed in Table 3, respectively. It can be found that dislocation strengthening and grain boundary strengthening were much more effective than other strengthening mechanisms. In Figure 13, we compare the results of theoretical calculations and experimental measurements. The discrepancy in the results can be attributed to a couple of reasons. First of all, some parameters used for the calculation were approximations or cited from other HEAs. Secondly, the distribution and shape of the B2 phase produced different resistance to plastic deformation, which is uncontrollable in the current LMD technology and requires further research. Thirdly, texture was formed in the LMD process, which also affected the strength of the material to a certain extent. In general, theoretical calculations were in good agreement with the experimental measurements, especially for grain boundary strengthening and dislocation strengthening, which played a remarkably important role in the LMD process.

In sum, the process conditions in the LMD technology provide several fine grains and a large number of dislocations for the metal; in this case, grain boundary strengthening and dislocation strengthening play an important role. However, as the laser power increased, the columnar crystals gradually grew, and the effect of grain boundary strengthening gradually weakened. Moreover, the Al_0.5_FeCoCrNi HEA had a large amount of lattice distortion, which caused solid solution strengthening. Furthermore, the FCC phase had a comparatively higher ductility, and the B2 phase had a higher strength. The designed Al_0.5_FeCoCrNi HEA combined the advantages of FCC and B2 structures, thereby exhibiting a good combination of strength and ductility. The two phases interacted at their interfaces, thereby restraining the deformation and relative movement, and further improving the mechanical properties of the Al_0.5_FeCoCrNi HEA. However, the change in laser power altered the content of the B2 phase, leading to differences in the mechanical properties for the materials manufactured by LMD. 

## 4. Conclusions

In this research, the LMD technology was used to fabricate Al_0.5_FeCoCrNi HEAs with varied laser powers. The microstructures, mechanical properties, and strengthening mechanisms were analyzed and compared. The main conclusions are as follows:

1. The Al_0.5_FeCoCrNi HEA produced by LMD exhibited an FCC + B2 dual-phase structure, and the FCC phase was dominant. With the increase in laser power, the fraction of the B2 phase gradually decreased. At the 1000 W bottom wall, the B2 phase content was the highest, accounting for 16.5%, and it gradually decreased with the deposition height of the wall. The B2 phase content at the 1800 W top wall was only 2.1%, which was the lowest.
2. The microstructure of the Al_0.5_FeCoCrNi HEA walls manufactured by LMD mainly consisted of columnar crystals. With increased laser power and wall height, the grains gradually grew and became much longer. The largest columnar crystals appeared at the 1800 W top wall with a size of 34.88 μm. The smallest columnar crystals appeared at the 1000 W bottom wall with a size of 14.31 μm.
3. The ultimate tensile strength and the ductility of the 1000 W bottom wall were 737 MPa and 24.6%, respectively, while those of 1800 W top wall were 641 MPa and 27.6%, respectively, demonstrating that the tensile strength of the walls decreased and the ductility increased with the increase in laser power. Because of heat accumulation, there were also significant differences in the mechanical properties at different locations. At the bottom, middle, and top of the 1800 W wall, the ultimate tensile strengths were 703 MPa, 676 MPa, and 641 MPa, respectively, and the ductility was 25.8%, 26.8%, and 27.6%, respectively. The strength of the bottom locations was better than that of the top locations, and the loss of ductility was not large.
4. Quantitative evaluation of the strengthening mechanisms, including solid solution strengthening, dislocation strengthening, and grain boundary strengthening, was carried out. It revealed that dislocation strengthening and grain boundary strengthening were the predominant influence mechanisms.
5. In industry, we can manufacture high-strength Al_0.5_FeCoCrNi HEAs by reducing laser power and preventing heat accumulation. Instead, high-ductility Al_0.5_FeCoCrNi HEA can be made, which can reduce cost and improve application value.

## Figures and Tables

**Table 1:** Chemical composition of 316 stainless steel (wt.%).

Chemical Element	Si	Mn	Cr	Ni	Mo	C	Fe
wt.%	0.63	1.19	17.99	12.84	2.56	0.07	Bal.

**Table 2:** Chemical composition of Al_0.5_FeCoCrNi (wt.%).

Chemical Element	Al	Fe	Cr	Ni	Co
Weight ratio	5.81	23.17	22.26	24.41	Bal.

**Table 3:** Various strengthening mechanisms identified in LMD-ed Al_0.5_FeCoCrNi HEA.

**Mechanism (MPa)**	$\sigma_{0}$	$\sigma_{SS}$	$\sigma_{GB}$	$\sigma_{DS}$	$\sigma_{y}$ (by Calculation)	$\sigma_{y}$ (by Experiment)
1000 W bottom	95	44.6	170.6	257.1	567.3.	431
1000 W middle	95	44.6	114.5	194.5	448.6	395
1000 W top	95	44.6	99.7	139.9	379.3	372
1400 W bottom	95	44.6	164.6	172.8	476.9	412
1400 W middle	95	44.6	101.6	155.7	396.9	372
1400 W top	95	44.6	90.3	152.9	382.8	368
1800 W bottom	95	44.6	108.1	166.8	414.5	391
1800 W middle	95	44.6	85.9	153.6	379.1	367
1800 W top	95	44.6	82.5	130.3	352.4	357

**Table 4:** The dislocation density ρ in the FCC phase and B2 phase.

Dislocation Density	$\rho_{FCC}$ $\;(m^{-2})$	$\rho_{B2}$ $\;(m^{-2})$
1000 W bottom	2.367 × 10^14^	7.106 × 10^14^
1000 W middle	1.981 × 10^14^	4.648 × 10^14^
1000 W top	1.406 × 10^14^	6.725 × 10^13^
1400 W bottom	1.960 × 10^14^	8.970 × 10^13^
1400 W middle	1.837 × 10^14^	3.384 × 10^13^
1400 W top	1.600 × 10^14^	2.183 × 10^13^
1800 W bottom	2.276 × 10^14^	3.402 × 10^13^
1800 W middle	1.809 × 10^14^	1.451 × 10^13^
1800 W top	1.313 × 10^14^	1.861 × 10^12^

## Data Availability

Not applicable.
